# Supplementary material for: Praziquantel activates a native cation current in Schistosoma mansoni
Source: Front Parasitol. 2023 Nov 16;2:1285177. doi: 10.3389/fpara.2023.1285177 (PMC11732042; doi:10.3389/fpara.2023.1285177)
Supplement: Supplementary file 1 [file Image_1.pdf]

Supplementary Information, Chulkov *et al*.

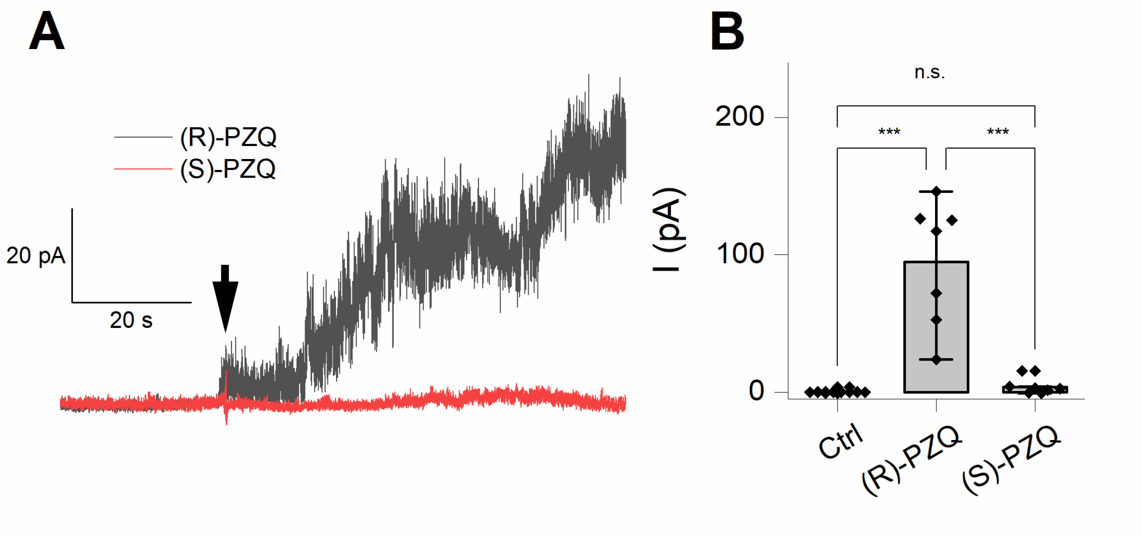

Supplementary Figure 1. Effect of PZQ enantiomers on the native response to PZQ.
(**A**) Representative recording from an adult male *S. mansoni* anterior neuron prior to, and after, addition of either (*R*)-PZQ or (*S*)-PZQ (10μM, arrow). (**B**) Cumulative measurements of neuronal current amplitude (mean±SE) before and after addition of (*R*)-PZQ or (*S*)-PZQ to the bath solution. ***$p \leq 0.001$, $n \geq 6$.
